# Genome analysis of poplar LRR-RLP gene clusters reveals RISP, a defense-related gene coding a candidate endogenous peptide elicitor

**DOI:** 10.3389/fpls.2014.00111

**Published:** 2014-03-28

**Authors:** Benjamin Petre, Stéphane Hacquard, Sébastien Duplessis, Nicolas Rouhier

**Affiliations:** ^1^INRA, Interactions Arbres/Microorganismes, UMR 1136Champenoux, France; ^2^Université de Lorraine, Interactions Arbres/Microorganismes, UMR 1136Vandoeuvre-lès-Nancy, France

**Keywords:** gene clustering, immunity, ligands, receptors, poplar, wounding

## Abstract

In plants, cell-surface receptors control immunity and development through the recognition of extracellular ligands. Leucine-rich repeat receptor-like proteins (LRR-RLPs) constitute a large multigene family of cell-surface receptors. Although this family has been intensively studied, a limited number of ligands has been identified so far, mostly because methods used for their identification and characterization are complex and fastidious. In this study, we combined genome and transcriptome analyses to describe the *LRR-RLP* gene family in the model tree poplar (*Populus trichocarpa)*. In total, 82 *LRR-RLP* genes have been identified in *P. trichocarpa* genome, among which 66 are organized in clusters of up to seven members. In these clusters, *LRR-RLP* genes are interspersed by orphan, poplar-specific genes encoding small proteins of unknown function (SPUFs). In particular, the nine largest clusters of *LRR-RLP* genes (47 *LRR-RLPs*) include 71 *SPUF* genes that account for 59% of the non-*LRR-RLP* gene content within these clusters. Forty-four *LRR-RLP* and 55 *SPUF* genes are expressed in poplar leaves, mostly at low levels, except for members of some clusters that show higher and sometimes coordinated expression levels. Notably, wounding of poplar leaves strongly induced the expression of a defense *SPUF* gene named *Rust-Induced Secreted protein* (*RISP)* that has been previously reported as a marker of poplar defense responses. Interestingly, we show that the *RISP-associated LRR-RLP* gene is highly expressed in poplar leaves and slightly induced by wounding. Both gene promoters share a highly conserved region of ~300 nucleotides. This led us to hypothesize that the corresponding pair of proteins could be involved in poplar immunity, possibly as a ligand/receptor couple. In conclusion, we speculate that some poplar SPUFs, such as RISP, represent candidate endogenous peptide ligands of the associated LRR-RLPs and we discuss how to investigate further this hypothesis.

## Introduction

Plants possess cell-surface receptors able to recognize extracellular molecules for subsequent intracellular signaling (Monaghan and Zipfel, 2012). Cell-surface receptors are organized into distinct families, which present a common architecture with a cytoplasmic part, a transmembrane domain and an extracellular part. Cytoplasmic domains allow distinguishing the two major classes of receptors: the receptor-like kinases (RLKs, with a cytoplasmic kinase domain) and the receptor-like proteins (RLPs, with a short cytoplasmic tail). In order to trigger signaling, RLKs use their own kinase domain, whereas RLPs likely require association with RLKs at the plasma membrane (Liebrand et al., 2013). A variety of extracellular domains, which directly interact with apoplastic ligands, have been described; the most common being composed of leucine-rich repeats (LRRs) (Monaghan and Zipfel, 2012; Sun et al., 2013). RLKs and RLPs with extracellular LRRs are abbreviated LRR-RLKs and LRR-RLPs, respectively.

LRR-RLPs evolved as a multigene family in higher plants. For instance, more than 50 *LRR-RLP* genes have been identified in the model plant *Arabidopsis thaliana* (Fritz-Laylin et al., 2005). Despite extensive genetic studies, the function of nearly all *A. thaliana* LRR-RLPs remains unknown (Wang and Fiers, 2010). The few plant LRR-RLPs characterized so far are able to perceive peptide ligands and trigger signaling cascades that control immunity or development. For example, tomato Cf-9 and Ve1 LRR-RLPs trigger plant immunity after the recognition of the fungal proteins Avr9 and Ave1, respectively (Jones et al., 1994; de Jonge et al., 2012). In contrast, *A. thaliana* CLV2 and TMM modulate plant development, respectively meristematic cell differentiation and leaf stomatal patterning (Jeong et al., 1999; Nadeau and Sack, 2002). CLV2 achieves its function through the recognition of endogenous peptides from the CLE family (Murphy et al., 2012).

Besides CLE peptides, several families of cell-surface receptor ligands are small endogenous peptides (~10–20 amino acids), which are often cleaved from larger precursor proteins of unknown function (~100–200 amino acids) and released into the apoplast where they function as messengers (Butenko et al., 2009). Among these endogenous peptides, those that are able to induce immune responses are commonly referred to as endogenous peptide elicitors (Ryan and Pearce, 2003; Yamaguchi and Huffaker, 2011). For instance, the *A. thaliana* plant elicitor peptide (AtPep) family is constituted of small peptides (~23 amino acids) encoded by larger pro-AtPep precursor proteins of ~100 amino acids (Huffaker et al., 2006). Members of the AtPep family bind to PEPR cell-surface receptors from the LRR-RLK family to trigger immune responses (Krol et al., 2010).

Here, we report the genome-wide analysis of the *LRR-RLP* gene family in the model tree species poplar. We observed that *LRR-RLP* genes are frequently associated with genes coding orphan, species-specific small proteins of unknown function (SPUFs). Moreover, transcriptomic data highlighted a coordinated expression for a pair of physically associated *LRR-RLP* and *SPUF* genes in response to wounding, suggesting that the gene products might be functionally linked.

## Results

### The poplar *LRR-RLP* gene family evolved recently by tandem duplication

**Figure 1:**
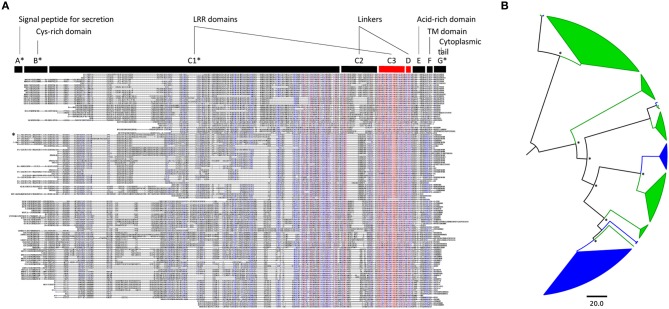
**Poplar and arabidopsis LRR-RLP families evolved independently. (A)** Alignment of the protein sequences of the 82 and 45 LRR-RLP from poplar and *A. thaliana*, respectively. Blue and red colors indicate a minimum of 50 and 90% of amino acid identity per position, respectively. The canonical domains of tomato Cf-9-like LRR-RLPs described by Fritz-Laylin et al. (2005) are indicated above the alignment. The asterisks mark the domains that are variable in size and position within the alignment; they have been arbitrarily adjusted to correspond to the sequence of poplar LRR-RLP9 (see text). The C3 and D domains, conserved in all sequences, are indicated in red and have been used for performing the phylogenetic analysis presented in **(B)**. LRR, Leucine-Rich Repeat; Cys, Cysteine; TM, Trans-Membrane. **(B)** Phylogenetic tree of poplar and *A. thaliana* LRR-RLP families. The analysis was done with the C3-D domains presented in **(A)**. Poplar sequences are highlighted in green, whereas arabidopsis sequences are in blue. Main nodes with Approximate Likelihood-Ratio Test (aLRT) values superior to 0.7 are marked with an asterisk.

**Figure 2:**
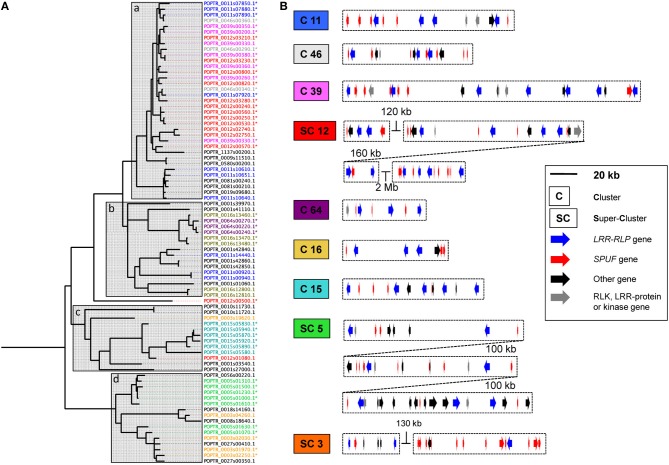
**Poplar *LRR-RLP* genes cluster with genes coding small-proteins of unknown function. (A)** Phylogenetic tree of the 82 LRR-RLP from poplar constructed with the C3-D domains. Almost all sequences gather into four main clades (a, b, c, and d) indicated by gray boxes. IDs have been colored according to their presence on similar chromosomes (chr.) or scaffolds (scf.), as follow: chr.3: orange; chr.5: green; chr.11: blue; chr.12: red; chr.15: cyan; chr.16: dark-yellow; scf.39: pink; scf.46: gray; scf.64: purple; any other chr. or scf.: black. Sequence IDs marked with an asterisk indicate that the corresponding gene model is represented in clusters depicted in **(B)**. **(B)** The nine main clusters (C) or super-clusters (SC) of *LRR-RLP* genes are depicted, approximately facing their corresponding phylogroup in **(A)**. All genes present within LRR-RLP clusters (i.e., any genes at less than 25 kb of an LRR-RLP gene) are represented. This threshold has been extended to 50 kb in the case of SC3.

A total of 82 *LRR-RLP* genes were identified in the poplar genome (Table 1; Table S1A). This value is in the range of those reported in *A. thaliana* and rice, 57 and 90, respectively (Fritz-Laylin et al., 2005; Wang et al., 2008). LRR-RLPs are composed of several domains numbered from A to G (Fritz-Laylin et al., 2005). Domains such as the LRR domain C1 or the linker domain C2 have a variable number of repeats. In contrast, the LRR domain C3 and the linker domain D are conserved and are thus suitable for sequence comparison and phylogenetic analyses (Figure 1A). A phylogenetic tree was inferred with the C3 and D domains of poplar and *A. thaliana* LRR-RLPs (Figure 1B). Sequences from each species gather within a few well-separated clades, highlighting the strong divergence of the two families and their probable independent evolution in poplar and *A. thaliana*. A phylogenetic tree focusing only on poplar LRR-RLPs revealed that 81 of the 82 sequences group into four distinct clades termed a, b, c, and d (Figure 2A). *LRR-RLP* genomic organization correlates with phylogeny, since genes that cluster in the genome sequence gather in the same phylogenetic clade. For instance, the six *LRR-RLPs* from chromosome 15 are all grouped into the clade c, whereas the seven *LRR-RLPs* from chromosome 5 are found in the clade d. A more accurate analysis of gene positions revealed that 66 of the 82 *LRR-RLPs* are organized in 23 clusters or super-clusters (Table 1). A cluster is constituted by at least two genes within a 50 kb stretch, whereas a super-cluster refers to a group of at least two clusters separated by less than 2 Mb. The nine largest clusters or super-clusters gathering 47 *LRR-RLPs* (57% of the family) are depicted in Figure 2B.

**Table 1:** **LRR-RLP and associated SPUF genes in *P. trichocarpa* and *A. thaliana***.

	***P. trichocarpa***	***A. thaliana***
*LRR-RLP* genes	82	45
*SPUF* genes	87	38
*LRR-RLP*-associated genes [avg. per LRR-RLP gene]	375 [4.75 ± 2.1]	597 [13.3 ± 3.7]
% of *SPUF* genes compared with the total no. of *LRR*-*RLP*-associated genes	23.2	6.4
% of *SPUF* genes in the whole predicted proteome	~20	~13
*LRR-RLP* genes clustered [no. of clusters]	66 [23]	29 [11]
SPUFs predicted secreted	10	10
SPUFs with no paralog	31	21
SPUFs with 1 or 2 paralogs	20	11
SPUFs with no homolog in other plants	34	29

### Poplar *LRR-RLP* genes are closely associated with genes encoding orphan, species-specific small proteins of unknown function (SPUFs)

We investigated carefully all *LRR-RLP*-associated genes (i.e., genes present within the 25 kb upstream or downstream of a given *LRR-RLP* gene) in the poplar genome sequence, and found a total of 375 *LRR-RLP*-associated genes (Table 1). Among these 375 genes, 87 (23% of all *LRR-RLP*-associated genes) encode small proteins (less than 200 amino acids) of unknown function that we abbreviated SPUF (Table 1). This value is close to the average percentage of SPUF (20%) found within the predicted proteome of *P. trichocarpa* (Table 1). However, within the nine biggest clusters of *LRR-RLPs* depicted in Figure 2, and without considering the *LRR-RLPs*, *SPUF* genes account for 59% of the gene content. Such an abundance of *SPUF* genes within poplar *LRR-RLP* clusters prompted us to further investigate them.

**Figure 3:**
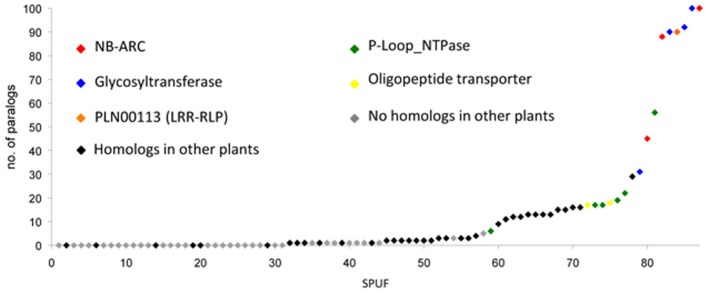
**Poplar SPUFs have limited paralogs and homologs in other plants**. Poplar SPUF sequences were used for homology searches against the predicted poplar proteome on the Phytozome portal, as well as the non-redundant protein database at the NCBI website. The dataset used, homology search details and homologs identified in other plants are detailed in Table S1B.

Using SPUF sequences as queries, we performed homology searches against the non-redundant protein database at NCBI. This analysis revealed that 53 SPUFs are poplar-specific whereas the remaining 34 have low homology with proteins from other plant species (Table S1B). Due to the presence of partially conserved or truncated domains (e.g., NB-ARC, glycosyl hydrolase, …), a dozen of SPUFs have numerous paralogs and homologs in databases (Figure 3). To estimate SPUF diversity within the poplar genome, we also performed homology searches against the predicted proteome of *P. trichocarpa* and found that 30 of the 87 *SPUFs* have no paralog and 21 have only one or two paralogs (Figure 3; Table 1). Altogether, these results show that many *SPUFs* are orphan and poplar-specific genes.

**Figure 4:**
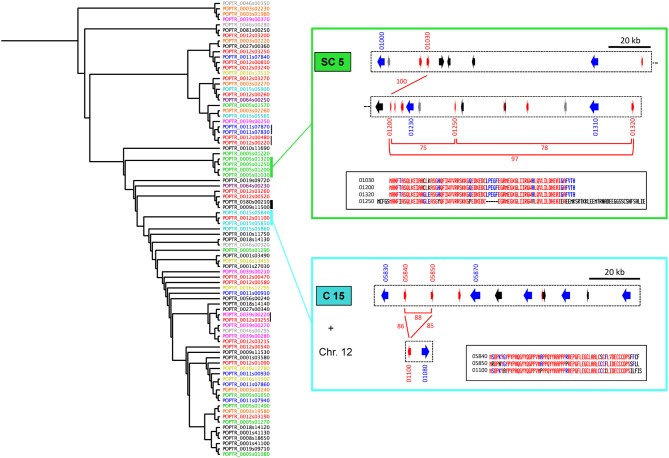
**Some families of SPUFs are gathered into the same LRR-RLP clusters**. The left-hand part of the figure presents a phylogenetic tree computed from poplar SPUF sequences. Sequence IDs of SPUFs are colored as their clustered LRR-RLPs in Figure 2. Vertical bars indicate close sequences for which gene models are physically associated within LRR-RLP clusters. Thick bars indicate examples presented in details in Figure 5D (black bar) or on the right-hand part of the figure (green and cyan bars). On the right-hand of the figure, the green rectangle presents a sub-part of Super-Cluster 5 (SC5), whereas the cyan rectangle presents the Cluster 15 (C15) as well as a *SPUF/LRR-RLP* pair from chromosome 12 (Chr. 12). The color code for arrows is as described in Figure 2. The last numbers of gene model IDs are indicated. Red bars and corresponding numbers indicate the percentage of amino acid identity between the protein products of the *SPUF* genes. The inserts in the black rectangles present SPUF alignments. Blue and red colors indicate a minimum of 50 and 90% of amino acid identity per position, respectively.

Interestingly, some *SPUF* paralogs are also physically associated within *LRR-RLP* clusters, strongly suggesting that they could have been duplicated conjointly with *LRR-RLP* genes. For example, a family of four related *SPUFs* are all grouped within the super-cluster 5 (Figure 4). Another family of three closely related *SPUFs* is dispatched between cluster 15 (two members) and chromosome 12 (one member, nearby a non-clustered *LRR-RLP*). Both the *SPUF* and the *LRR-RLP* from chromosome 12 are the closest paralogs of *SPUFs* and *LRR-RLPs* from the cluster 15 (Figures 2, 4). In this case, the *LRR-RLP*/*SPUF* pairs from chromosomes 15 and 12 likely result from a recent event of duplication and transposition. This point is supported by the fact that chromosomes 12 and 15 are “twin chromosomes,” resulting from the recent whole genome duplication in poplar (Tuskan et al., 2006).

Noteworthy, 10 SPUFs possess an N-terminal signal peptide predicted to target the proteins to the secretory pathway (Table 1). The gene coding one of these proteins has been previously reported as the most induced gene in poplar leaves during resistance to the leaf rust fungus *Melampsora larici-populina* (Rinaldi et al., 2007). The corresponding protein, composed of 60 amino acids in its predicted mature form, was named Rust-Induced Secreted Protein (RISP). RISP has no known function and no homolog in other plants, and has been speculated to be a novel component of the poplar immune system.

Besides *SPUF* genes, 30 genes coding proteins with a restricted number of LRR-domains have been found in the vicinity of poplar *LRR-RLP* genes (in gray in Figure 2B; Table S1A). It is tempting to consider them as reservoirs of diversity or relics of *LRR-RLP* gene evolution, although we cannot completely exclude that these are mis-annotated or pseudo-genes. Among other notable *LRR-RLP*-associated genes identified within *RLP-LRR* clusters are RLKs and serine-threonine or lectin kinases (Table S1A), which may eventually be part of the same signaling cascades.

### Some clusters of *LRR-RLP* and *SPUF* genes are expressed in poplar leaves

**Figure 5:**
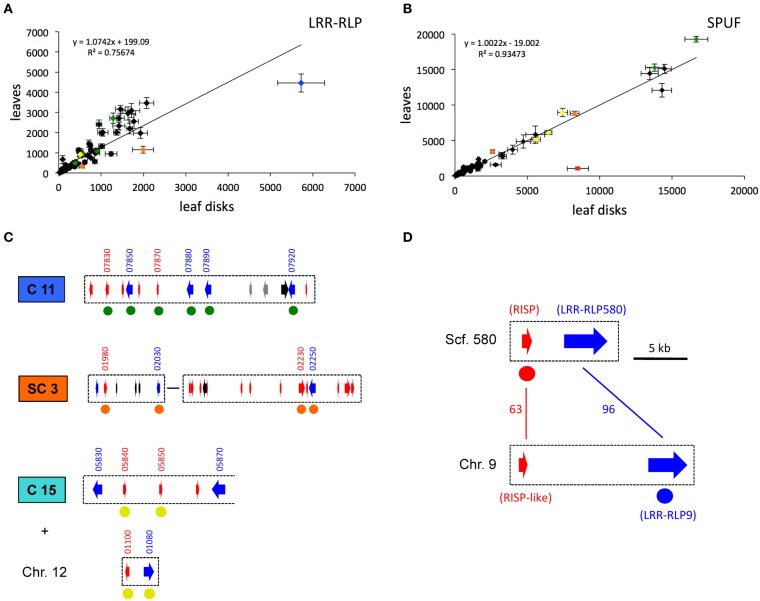
**The gene expression of some pairs of *LRR-RLP/SPUF* genes is coordinated. (A,B)** The average levels of gene expression measured in poplar leaves (non-wounded condition) and in excised leaf disks (wounded condition) with oligoarrays are compared for the considered *LRR-RLP* **(A)** and *SPUF* **(B)** genes. Error bar: SE, *n* = 6. The linear regression and associated values are indicated for each graph. Colored points are further discussed in the text and in the parts **(C,D)**. The complete transcriptome dataset is available in Table S1C. **(C)** Cluster 11 (C11), a sub-part of Super-Cluster 5 (SC5), and Cluster 15 (C15) associated with a pair of LRR-RLP/SPUF genes from chromosome 12 (Chr.12) are depicted (for color code and gene model IDs, see Figure 2 and the legend of Figure 4). Green (C11), orange (SC5), and yellow (C15 + Chr.12) dots under gene models indicate a co-expression of LRR-RLP and SPUF genes in poplar leaves, as shown in **(A,B)**. **(D)** *RISP* and *LRR-RLP* genes from scaffold 580 (Phytozome ID Poptr_0580s00210 and Poptr_0580s00200, respectively), are the closest paralogs of *RISP-Like* and *LRR-RLP9* genes from chromosome 9 (Phytozome ID Poptr_0009s11500 and Poptr_0009s11510), respectively. Bars and associated numbers indicate the percentage of amino acid identity. Red and blue dots under RISP and LRR-RLP9 genes indicate their co-induction as shown in **(A,B)**.

The expression of the 82 *LRR-RLP* genes and of 82 of the 87 *SPUF* genes was examined in mature poplar leaves using whole-genome poplar oligoarrays (Table S1C). Overall, 44 *LRR-RLPs* were expressed above the background threshold, but at low to medium levels (<4000) (Figure 5A). POPTR_0009s11510, the only LRR-RLP present on chromosome 9 (hereafter termed LRR-RLP9), is the only one that presented a slightly higher expression in poplar leaves (>4000) (Figure 5A). Among the *SPUFs*, 55 presented a detectable expression, and five of them showed a very high expression level (>10,000) (Figure 5B). Interestingly, some specific clusters gathering several pairs of *LRR-RLP* and *SPUF* genes showed medium to high expression levels in leaves. For example, the four *LRR-RLPs* and two *SPUFs* from cluster 11 are concomitantly expressed in leaves (Figure 5C). Similarly, two pairs of *LRR-RLP*/*SPUF* from the super-cluster 3 are co-expressed. Interestingly, the three *SPUFs* dispatched between chromosome 15 and 12 (highlighted previously, see Figure 4) were among the most highly expressed *SPUF* genes in leaves, along with the nearby *LRR-RLP* from chromosome 12 (Figure 5C).

To extend our survey of *LRR-RLP* and *SPUF* gene expression, we explored the PopGenIE portal (http://popgenie.org/), which compiles expression data from rust-infected leaves, mature leaves, leaf disks, seeds, stems and suspension cells from poplar. Expression data were available for 47 *LRR-RLP* and 36 *SPUF* genes. A large majority (42 *LRR-RLP*s and 34 *SPUF*s) was expressed at low levels and did not exhibit any particular expression profile or obvious regulation in the analyzed conditions (Figure S1). Nevertheless, five *LRR-RLPs* and two *SPUFs* showed interesting profiles. Indeed, the POPTR_0005s01490 and POPTR_0011s07870 *SPUF* genes presented a high expression in stems and rust-infected leaves, respectively, whereas the five *LRR-RLPs*, including the above-mentioned LRR-RLP9, presented a high expression during stress treatments (rust-infected leaves and/or leaf discs).

### RISP is strongly induced in poplar leaves upon wounding

Most genes involved in immune responses (including cell-surface receptors and precursors of endogenous peptide elicitors) have an inducible expression in response to stresses like wounding or pathogen attack (Kemmerling et al., 2011; Yamaguchi and Huffaker, 2011). In order to identify poplar *LRR-RLP* and *SPUF* genes that likely participate to defense mechanisms, we compared transcript expression profiles between excised poplar leaf discs and entire leaves (i.e., wounded vs. non-wounded leaves) by using whole-genome poplar oligoarrays. As expected, the expression of genes classically associated with wounding such as wound-response proteins, chitinases, or protease inhibitors were induced in leaf discs (Figure S2; Table S1C). The expression levels of all *SPUFs* remained very stable after wounding, except one, the *RISP* gene, which was strongly induced (~10 fold) (Figure 5B). This observation supports the above-mentioned statement that RISP could be involved in poplar defense responses (Rinaldi et al., 2007). Similarly, almost all *LRR-RLPs* remained stably expressed in both conditions, except two (POPTR_0003s02030 and LRR-RLP9) that were slightly (<2 fold) induced by wounding (Figure 5A). Interestingly, the predicted LRR-RLP9 gene is a very close paralog (>96% identity at the amino acid level) of POPTR_0580s00200 (hereafter LRR-RLP580), the *LRR-RLP* gene associated with the *RISP* gene. The co-regulation of both RISP and LRR_RLP9 genes observed by these oligoarray data has been further confirmed by a RNA-Seq analysis performed over a time-course infection of poplar leaves with virulent and avirulent strains of *M. larici-populina*. First, we observed a clear co-induction at 6 hpi, which likely correspond to a wounding response due to the leaf detaching (Figure S3). Second, we confirmed the strong and specific induction of RISP during poplar defense responses against an avirulent strain of *M. larici-populina* as previously observed (Rinaldi et al., 2007). In this case, the expression of LRR-RLP9 was only slightly induced (Figure S3).

Next, the promoter regions of both *RISP* and *LRR-RLP9* genes have been analyzed to identify putative common regulatory elements explaining the observed co-expression. Whereas we did not detect particular signature or conservation in the 0 to −700 nucleotide region, we identified a stretch of ~300 bp with almost 90% nucleotide identity in the −700 to −1000 region (Figure S4). This striking conservation between the promoter regions of two unrelated genes may well explain the observed co-expression and will deserve future analysis.

### *RISP* gene is physically associated with a chimera of *LRR-RLP9* and *LRR-RLP580*

**Figure 6:**
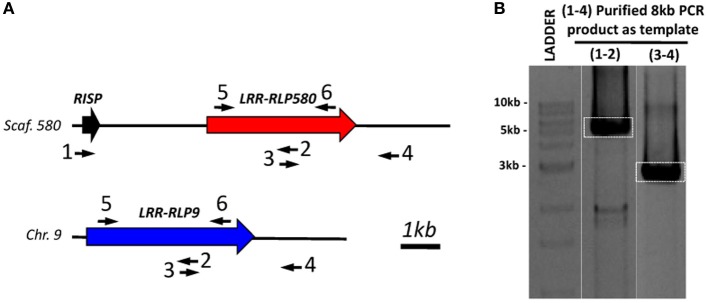
**Physical association between *RISP* and a chimera of *LRR-RLP580* and *LRR-RLP9*. (A)** Schematic representation of the location of *RISP*, *LRR-RLP580*, and *LRR-RLP9* on *P. trichocarpa* genomic sequence (Phytozome v2.2). The primers used to amplify and sequence the fragment containing the *LRR-RLP* associated with *RISP* are indicated. Note that the primer 1 is specific to the *RISP* gene whereas primers 2, 3, and 4 could not discriminate between *LRR-RLP9* (blue) and *LRR-RLP580* (red) genes. The primers 5 and 6 were designed to amplify the extracellular domain of both *LRR-RLP9* and *LRR-RLP580*. **(B)** The large 8 kb fragment amplified using the 1–4 primer pairs was used as template to amplify the two shorter fragments (using 1–2 and 3–4 primer pairs) that were used for sequencing.

The only paralog of RISP is POPTR_0009s11500 (hereafter termed RISP-like). RISP and RISP-like proteins show 63% identity and both carry a conserved predicted signal peptide (Figure 5D). In the current genome assembly, the *RISP-like* gene is adjacent to *LRR-RLP9* gene onto chromosome 9. Hence, two pairs of paralogous genes (*RISP*/*LRR-RLP580* and *RISP-like*/*LRR-RLP9)* seem to be present at different positions in the poplar genome (Figure 5D). Considering that the *RISP* and *LRR-RLP580* genes are located onto the small (~13 kb), unresolved scaffold 580, a poorly assembled part of poplar genome, we sought to investigate further the physical association between *RISP* and *LRR-RLP580* by using a combination of PCR amplification and sequencing approaches (Figures 5D, 6A). Using *P. trichocarpa* genomic DNA as a template, we successfully amplified a major 8 kb fragment using a forward primer specific to the *RISP* gene (primer 1) and a reverse primer that cannot discriminate between the regions downstream the *LRR-RLP9* and *LRR-RLP580* genes (primer 4), both sequences being too similar (Figure 6B). By using additional primers targeting the coding sequence of both *LRR-RLPs* (primers 3 and 4), we amplified, from the 8 kb PCR product as a template, two fragments of ~5 and 3 kb, respectively, (Figure 6B). Sequencing of these fragments unambiguously confirmed the presence of the *RISP* gene, whereas the sequenced portion of the associated *LRR-RLP* was a chimera of *LRR-RLP9* and *LRR-RLP580*, likely indicating that the current genome assembly is not correct at this particular locus. This chimeric organization was further confirmed by amplifying the sequence coding the extracellular part of both *LRR-RLP* genes from cDNA, using primers 5 and 6. The sequencing of the cloned product revealed that the expressed sequence was indeed a combination of both predicted genes (Figure S5). Altogether, these observations indicated that *LRR-RLP9* and *LRR-RLP580* genes are mis-predicted and that they likely constitute a single gene that is physically associated and co-regulated with the *RISP* gene in response to wounding.

## Discussion

In this study, by analysing the LRR-RLP gene family in poplar, we observed that *LRR-RLP* genes are arranged into large clusters which are interspaced by genes coding SPUFs. Based on the observed co-regulation upon wounding of RISP and its physically associated LRR-RLP and on the presence of similar regulatory elements in their promoter regions, we hypothesize that both gene products may be functionally linked and by extension we suggest that some SPUFs could represent candidate endogenous ligands of their genome associated LRR-RLPs. Below, we did not discuss the genomic organization of LRR-RLP in poplar as this is not different from other plant species, but we rather discuss how to validate LRR-RLP/SPUF interactions and whether such genomic analysis can be extended to other species.

### Do *SPUF* genes encode candidate endogenous ligands of LRR-RLPs?

In prokaryotes, genes that are functionally related are often gathered in operons and are co-regulated. Although such organization is less prominent in eukaryotes, a recent review illustrated the presence of operon-like clusters of secondary metabolism genes in plants, suggesting that physical association of genes can indeed reflect functional links between gene products (Kliebenstein and Osbourn, 2012). Besides, in tomato, the *Prf* gene clusters with several *Pto* resistance genes and it has been shown that the proteins physically interact (Rosebrock et al., 2007). Hence, operon-like clusters of genes coding interacting proteins involved in the immune response exist in plants. Nevertheless, physical associations of genes encoding a cell-surface receptor and its endogenous ligand have not been reported in plants so far. This may have been hampered by the limited number of identified receptor/ligand pairs or by the fact that the genes are not systematically associated in all plant genomes. Concerning the first point, genes coding characterized receptor/ligand pairs such as PEPRs/AtPeps or CLV2/CLE are not physically associated into the Arabidopsis genome. For the latter point, the existence of such physical association in one or in a few species only could have allowed in principle identifying functionally related receptor/ligand couples. However, the impossibility for example to unambiguously identify orthologs of AtPep, systemin, or CLV3 peptides in poplar genome suggests that such general inter-species comparisons could be precluded.

We showed that in poplar, *LRR-RLPs* cluster primarily with genes coding SPUFs. This physical association at the genomic level and the coordinated expression pattern of some *LRR-RLP*/*SPUF* pairs in some stress conditions suggest that they might function together, possibly as ligand/receptor couples. For instance, the physical association of *RISP* gene with an *LRR-RLP* gene is corroborated by the fact that both genes are induced, although slightly for the *LRR-RLP*, in response to wounding. Previous studies reported *RISP* induction in leaves during effective immune responses to an avirulent strain of the leaf rust pathogen *M. larici-populina* (Rinaldi et al., 2007). Also, comparison of RISP sequences between different poplar cultivars suggests an evolution under positive selection, a characteristic of genes involved in biotic interactions (Petre et al., 2012). Taken together, these data strongly suggest a role for RISP in poplar immunity. Preliminary studies with a purified recombinant RISP have shown its ability to trigger poplar cell-culture alkalinization, a hallmark of immune responses activation (Petre et al., unpublished data). Further studies should investigate if a direct interaction exists between selected SPUF/LRR-RLP pairs, but this remains technically challenging even in amenable plant species. Although recent studies showed that some cell-surface receptors are still functional when ectopically expressed in plants from different botanical families, such approach failed in most of the cases (Lacombe et al., 2010; de Jonge et al., 2012). Nevertheless, the recent finding that several LRR-RLPs require the LRR-RLK SOBIR1/EVR as a co-receptor to function may explain why transfer between different species did not work and may help overcoming this obstacle (Liebrand et al., 2013). We identified two very close SOBIR1/EVR homologs in poplar genome (POPTR_0015s09830 and POPTR_0012s09230, both 66% identity with SOBIR1/EVR), which are highly expressed in poplar leaves (Petre et al., 2012). However, although they are positioned on chromosomes 12 and 15, the two chromosomes with the highest *LRR-RLP* content, they are not included into the identified *LRR-RLP* clusters. Thus, possible future experiments could consist in the co-expression of poplar LRR-RLPs and poplar SOBIR1/EVR homologs in other amenable plants, followed by a treatment with the genome-associated purified SPUF and phenotyping.

### Does the analysis of genomic organization could help identifying LRR-RLP ligands in other plants?

Considering the obstacles encountered by past genetic approaches, our analysis could help accelerating the discovery of LRR-RLP functions in poplar and possibly in other model plant systems. In the *A. thaliana* genome, we identified 38 LRR-RLP-associated *SPUFs*, which present features similar to poplar *SPUFs* (i.e., orphan genes, species-specific). However, the density of *SPUF* genes in *A. thaliana LRR-RLP* clusters is lower compared with the average number of SPUFs in the predicted proteome (6 vs. 13%) (Table 1). Besides SPUFs, we identified some *LRR-RLP*-associated genes coding known peptide ligands in *A. thaliana* genome as rapid-alkalinization factors (RALF) and known defense-related secreted proteins (i.e., defensins) (Table S1D), which would be of interest for further investigations.

## Materials and methods

### Identification of *LRR-RLP* genes in the poplar genome, phylogeny, and sequence analyses

*LRR-RLP* genes have been identified in the *P. trichocarpa* “Nisqually-1” genome sequence, version 2.2, hosted on the Phytozome portal (http://www.phytozome.net/poplar) by amino acid sequence homology searches using tomato Cf-9 protein sequence as a query, then complemented with recursive searches with poplar LRR-RLP sequences representative from each clade (A–D). We considered only the *LRR-RLP* genes presenting the Cf-9 canonical domains and more than 15 LRRs (Fritz-Laylin et al., 2005). Among the 82 *LRR-RLP* genes retained for the analysis, only two required a manual correction due to intron mis-prediction (Table S1A, Text S1). For the phylogenetic analyses, we have retained the 45 strict Cf-9 homologs among the 57 *LRR-RLP* genes identified previously in *A. thaliana* (Wang et al., 2008). Only the conserved C3 and D domains (Fritz-Laylin et al., 2005) of LRR-RLP sequences and the whole SPUF sequences were used to build phylogenetic trees on the Phylogeny website (http://www.phylogeny.fr/), by using default parameters (Muscle alignment, Gblocks curation, PhyML phylogenetic tree, and SH-like approximate likelyhood–ratio test for branch support) (Dereeper et al., 2008). The trees were observed and edited in the FigTree v1.2.3 software (http://tree.bio.ed.ac.uk/software/figtree/). All protein-coding genes present in the 50 kb vicinity of poplar and *A. thaliana LRR-RLP* genes were inspected manually on the Phytozome portal. This limit was determined to maximize the genomic area investigated and to have a single *LRR-RLP* in the genome portion analyzed. All genes coding for a small protein of unknown function, defined as proteins with a size below 200 amino acids and with no annotation and no pfam available, were retained and designated as SPUF genes. Only 12 needed a manual annotation for start and stop codons (Table S1A). Signal peptides for secretion were predicted with SignalP 3.0 (http://www.cbs.dtu.dk/services/SignalP-3.0/) and SecretomeP 2.0 (http://www.cbs.dtu.dk/services/SecretomeP/) servers and amino acid sequence alignments were executed on the Multalin website (http://multalin.toulouse.inra.fr/multalin/). Poplar and *A. thaliana* SPUF paralogs have been searched in respective predicted proteomes by amino acid homology searches, whereas homologs in other plants as well as conserved domains have been searched in the non-redundant protein database at NCBI (Blastp, *E*-value < 10^−10^).

### Poplar transcript expression analysis

Poplar genome oligoarray data obtained from hybrid poplar “Beaupré” (*P. trichocarpa* × *P. deltoides*) leaf disks (i.e., wound treatment) collected at six different time-points from 18 to 48 h after inoculation with *M. larici-populina* (GSE39727 at NCBI GEO) were compared with strictly identical experiments performed on entire leaves (i.e., non-wounded treatment; GSE34802 at NCBI GEO). Poplar cultivation and inoculation procedures have been carried out as previously reported (Rinaldi et al., 2007). Isolation of total RNA, cDNA synthesis and oligoarrays were performed as previously described (Petre et al., 2012). The average of the six conditions in each dataset has been considered for quantitative comparison of gene expression with Microsoft Excel and R. Poplar oligoarray data are presented in Table S1C.

RNA-seq data of a time-course infection of poplar leaves with virulent and avirulent strains of *M. larici-populina* (same inoculum as in Rinaldi et al., 2007) used here to confirm RISP/ *LRR-RLP9* expression profiles (Figure S3) were produced in the framework of a different research project and will be published elsewhere.

### Genomic DNA isolation, PCR amplification, and sequencing

Genomic DNA was isolated using the DNeasy Plant Mini Kit (Qiagen, Courtaboeuf, France) from 100 mg of *P. trichocarpa* “Nisqually-1” leaves. To amplify the genomic DNA fragment containing the *RISP* gene and the associated *LRR-RLP*, specific primers were designed in the coding sequence of the *RISP* gene (primer 1: 5′AGTAGCAAACAAAGTTGCCACCCCAGTC3′) and in the downstream conserved sequence of both *LRR-RLP9* and *LRR-RLP580* (primer 4: 5′GAGATGCTAATGGGATGAGGTTT3′). The LongRange PCR Kit (Qiagen) was used to amplify an 8 kb fragment using 25 ng of *P. trichocarpa* genomic DNA as a template. Two additional primers (primer 2: 5′CATGCAAGTGGTTATTGCTCA3′ and primer 3: 5′TGAGCAATAACCACTTGCATG3′) were designed in the conserved coding sequence of both *LRR-RLPs* and two fragments (using the 1–2 and the 3–4 primer pairs) were amplified from 1 ng of the purified 8 kb PCR fragment using the LongRange PCR Kit. The amplified fragments were purified with the QIAquick PCR purification kit (Qiagen) and directly sequenced according to GenomeLab Dye terminator cycle sequencing with Quick Start kit (Beckman Coulter, Villepinte, France) on a Genetic Analysis System CEQ-8000 (Beckman Coulter). The sequence coding the extracellular domain of the chimera between LRR-RLP 580 and LRR-RLP 9 has been amplified from *P. trichocarpa* cDNAs using primer5: 5′ggggggCATATGTTGTCTTCAAATTTCTCCTCT3′; and primer6: 5′ggggggGGATCCTTATGCTTTCCATCCAAATCCATC3′ and cloned into the NdeI and BamHI restriction sites (underlined in the primers) of a pET28a vector before sequencing.

## Author's contribution

Benjamin Petre carried out the *in silico* analysis, transcriptome analyses, PCR cloning of the LRR-RLP extracellular part and drafted the manuscript. Stéphane Hacquard performed the PCR amplification/sequencing of the genomic fragment containing RISP and the LRR-RLP genes. Sébastien Duplessis performed the promoter analysis and contributed the unpublished RNA-Seq data. All authors contributed to the conceptual design of the experiments, participated in the writing of the manuscript, have read and approved the final manuscript.

### Conflict of interest statement

The authors declare that the research was conducted in the absence of any commercial or financial relationships that could be construed as a potential conflict of interest.
