# Supplementary material for: Genome analysis of poplar LRR-RLP gene clusters reveals RISP, a defense-related gene coding a candidate endogenous peptide elicitor
Source: Front Plant Sci. 2014 Mar 28;5:111. doi: 10.3389/fpls.2014.00111 (PMC3975113; doi:10.3389/fpls.2014.00111)
Supplement: Supplementary file 1 [file Presentation1.PDF]

SUPPLEMENTARY MATERIAL

Text S1. Conserved LRR-RLP domain (C3-D) used for phylogenetic analyses. The C3-D domain from LRR-RLP of poplar (82 sequences) and *A. thaliana* (45 sequences) used to build the phylogenetic tree depicted in Figure 1 are presented in fasta format.

```
>POPTR_0005s01000.1
DLSCNRFTGEIPTEWGNLSGIYSLNLSQNNLTGLIPSSFSNLKHIESLDLSHNNLNGRIPAQLVELTFLAV
FNVSYNNLSGRTPEMKNQFGTFDESSYKGNPLLCGPPLQNSC

>POPTR_0005s01500.1
DLSCNRFNGEIPTEWGNLSGIYSLNLSQNNLTGLIPSSFFNLKQIESLDLSHNNLNGRIPAQLVELTFLEV
FNVSYNNLSGRTPEMKNQFATFDESSYKGNPLLCGPPLQNSC

>POPTR_0005s01230.1
DLSCNRFTGEIPTEWGNLSGIYSLNLSQNNLTGLIPSSFSNLKHIESLDLSHNNLNGRIPAQLVELTFLAV
FNVSYNNLSGRTPEMKNQFGTFDESSYKGNPLLCGPPLQNSC

>POPTR_0005s01310.1
DLSCNRFTGEIPTEWGNLSGIYSLNLSQNNLTGLIPSSFFNLKQIESLDLSHNNLNGRIPAQLVELTFLAV
FNVSYNNFSGRTPEMKNQFATFDESSYKGNPLLCGPPLQDSC

>POPTR_0005s01630.1*
DLSSNNFLGAIPQELGSLSEIHALNLSHNNLAGSIPATFSNLKQIESLDVSHNNLNGRIPAQLIELTFLEV
FNVSYNNLSGKTPEMKYQFATFDESSYKGNPLLCGPPLQNSC

>POPTR_0005s01070.1
DLSCNNFLGAIPQELGNLCEIHALNLSHNNLVGSIPATFANLKQIESLDLSYNNLNGAIPQQLTEITTLAV
FSVAHNNLSGKTPERKYQFGTFDESSYEGNPFLCGPPLQNNC

>POPTR_0005s01610.1
DLSCNRFTGEIPTEWGNLSGIYALNLSQNNFNGLIPPSFSNLKQIESLDLSHNNLNGRIPAQLVELTFLAV
FNVSYNKLSGRTPEMKNQFATFDESSYKGNPLLCGPPLQNSC

>POPTR_0580s00200.1
DLSHNSFIGEIPESIGKLKALKQLNFSHNSLTGYIQPSLGNLANLESLDLSSNLLTGRIPMQLADLTFLSV
LNLSHNQLEGPIPKGKQFNTFNKGSFEGNSGLCGFQISKEC

>POPTR_0046s00340.1
DLSNNNFTGEIPKVIGKLKALQQLNLSHNSLIGHIQSSLEKLTNLESLDLSSNLLTGRIPTQLGDLTFLAI
LNLSHNQLEGPIPSGEQFNTFDASSFEGNLGLCGSQVLKQC

>POPTR_0046s00360.1
DLSKNNFTGEIPKVIGKLKALQQLNLSHNSLTGHIQSSLGNLTNLESLDLSSNLLTGRIPTQLGGLTFLAI
LNLSHNQLEGRIPSGEQFNTFNPSSFEGNLGLCGFQVLKEC

>POPTR_0046s00290.1
DLSNNNFTGEIPKVIGKLKALQQLNLSHNSLNGHIQSSLGNLTNLESLDLSSNLLTGRIPTQLGGLTFLAI
LNLSYNQLEGPIPSGEQFNTFDASSFEGNLGLCGSQVLKKC

>POPTR_0018s14160.1
DFSRNNFTGEIPPEIGNLSGIKVLNLSHNSLTGPIPPTFSNLKEIESLDLSYNKLDGEIPPRLTELFFLEF
FSVAHNNLSGKTPTRVAQFATFEESCYKENPFLCGEPLPKIC

>POPTR_0003s01970.1
```

```
DLSDNNFVGAIPPEFGNLSEILSLNLSHNNLTGSIPATFSNLKRIESLDLSYNNFNGDIPPQLTEMTTLEV
FSVAHNNLSGKTPERKYQFGTFDESCYEGNPFLCGPPLRNNC

>POPTR_0003s02250.1
DLSNNNFGGAIPQEFGNLSEIRSLNLSHNNPTESIPATFSNLKQIESLDLSYNNLNGVIPPQLTEITTLEV
FSVAHNNLSGWTPERKYQFGTFDESCYEGNPFLCGPPLRNNC

>POPTR_0003s04260.1
DFSCNNFIGEIPPEIGNLSMIKVLNLSHNSLTGPIPPTFSNLKEIESLDLSYNKLDGEIPPQLIELFSLEF
FSVAHNNLSGKTLARVAQFSTFEESCYKDNPFLCGEPLPKIC

>POPTR_0003s19620.1
DLSSNALCGEIPEEVTRLSELQSLNLSQNSLTGRIPEGIGSLRYLESMDFSVNQLSGEIPQSMSDLTFLSH
LNLSDNRLRGRIPSGTQLQSFGPSSFSGNELCGPPLSKNC

>POPTR_0003s02030.1
DLSNNNFVGAIPPEFGNLSAILSLNLSHNNLTGSIPATFSNLKHIESLDLSYNNLNGAIPPQLTEITTLEV
FSVAHNNLSGKTPERKYQFGTFDASCYKGNPFLCGTPLQNNC

>POPTR_0019s09680.1
DLSCNKFTGKIPESLRKLKSLKQLNLSHNSLIGCIQPSLGNLTDLESLDLSSNLLAGRIPQELVDLTFLQV
LNLSYNQLEGLIPQGKQFNTFENGSYEGNLGLCGFPLQVKC

>POPTR_0056s00220.1
DLSCNRFTGEIPTEWGNLSGIIALNLSQNNLTGLIPSSFSNLKQIESLDLSHNNLKGRIPTQLVELTFLAV
FNVSYNNLSGRTPEIKNQFATFDESSYKGNPLLCGPPLQNSC

>POPTR_0064s00240.1
DLSRNGFEGGIPEVLGDLKALHLLNLSNNFLSGGIPPSLSNLKKLEALDLSQNKLSGEIPVQLAQLTFLAV
FNVSHNFLSGRIPRGNQFETFDNTSFDANPALCGEPLSKEC

>POPTR_0064s00270.1
DLSSNGFEGGIPEVLGDLKALHLLNLSNNFLSGRIPPSLSNLKELEALDLSHNKLSGEIPVQLAQLTFLEI
FNVSHNFLSGPIPRGNQFGAFDSTSFDANSGLCGEPLSKKC

>POPTR_0064s00220.1
DLSSNGFEGGIPEVLGDLKALHLLNLSNNFLSGGIPPSLSNLKELEALDLSHNKLSGEIPVQLAQLTFLAV
FNVSHNFLSGRIPRGNQFETFDNTSFDANPGLCGEPLSKEC

>POPTR_0039s00300.1
DLSSNSFTGEIPKLIGKLKGLQQLNLSHNYLTGHIQSSLRILNNLESLDLSSNLLTGRIPIQLVDLTFLQV
LDLSHNRLEGPIPKGKQFNTFDHRSFEGNSGLCGFPMPEEC

>POPTR_0039s00260.1
DLSNNNFTEEIPKVIGKLKALQQLNLSHNSLAGYIQSSLGILTNLESLDLSSNLLTGRIPMQLGVLTFLAI
LNLSHNQLEGPIPSGKQFNTFNASSFEGNLGLCGFQVLKEC

>POPTR_0039s00200.1(ok)
DLSNNNFTGEIPKVIGMLKALQQLNLSHNSLTGHIQSSLENLTNLESLDLSSNLLTGRIPTQLGGLTFLAI
LNLSHNQLEGPIPSGEQFNTFDASSFEGNLGLCGSQVLKKC

>POPTR_0039s00350.1(ok)
DISDNNFSGPLPKVIGKLKALQQLNLSHNSLTGLTFLAILNLSHNHLEGPIPSGAQFNTFDASSFEGHLGL
CGSQVLKKC

>POPTR_0039s00330.1
DLSNNNFTGGIPKVIGKLKALQQLNLSHNSLTGHIQSSLGNLTNLESLDLSSNLLTGRIPTQLGGITFLAI
LNLSHNQLKGRIPCGEQFNTFTATSFEGNLGLCGFQVLKEC
```

>POPTR_0039s00380.1
DLSNNNFTGEIPKMIGKLKALQQLNLSHNSLTGQIQSSLGNLTNLESLDLSSNLLTGRIPTQLGGLTFLAI
LNLSHNQLEGRIPSGEQFNTFDASSFEGNLGLCGSQVLKKC

>POPTR_0039s00360.1
DLSNNNFTGEISKVIGKLKALQQLNLSHNSLTGHIQSSLENLTNLESLDLSSNLLTGRIPTQLGGLTFLAI
LNLSHNQLEGRIPSGGQFNTFTASSFEGNLGLCGFQVLKEC

>POPTR_0009s11510.1
DLSHNSFIGEIPESIGKLNALQQLNFSHNSLTGYIQPSLGNLANLESLDLSSNLLTGRIPMQLADLTFLSV
LNLSHNQLEGPIPKGKQFNTFNKGSFEGNSGLCGFQISKEC

>POPTR_0012s02750.1
DLSNNSFTGEIPKPIGKLKALQQLNLSHNFLTGHIQSSLGFLTNLQSLDMSSNMLTGRIPLQLTDLTFLEV
LNLSQNKLEGPIPGGKQFNTFDPSSFQGNLGLCGFPMPTEC

>POPTR_0012s02740.1
DLSKNSFTGEIPKPIGKLKGLQQLNLSHNFLTGHIQSSLGFLTNLQSLDMSSNMLTGRIPVQLTDLTFLQV
LNLSQNKLEGPIPVGMQFNTFDASSFQGNLGLCGIQVLTEC

>POPTR_0012s01080.1
DFSGNNLSGEIPEEITGLLELVALNLSGNNLTGVIPQKIDHLKLLESLDLSRNHFYGAIPLTMAALNFLSC
LNVSCNNLSGKIPSSTQLQSFDASAFTGNPALCGLPVTQKC

>POPTR_0012s00530.1
DFSNNNFIGEISKVIGKLKALQQLNLSHNSLTGHIQSSLEMLTYLESLDLSSNLLTGRIPVQLADLTFLGV
LNLSHNHLEGAIPIGKQFNTFNASSFEGNLGLCGFPMPKEC

>POPTR_0012s03230.1
DLSNNNFTGEIPKMIGKLKALQQLNLSHNSLTGQIQSSLGNLTNLESLDLSSNLLTGRIPTQLGGLTFLAI
LNLSHNQLEGRIPSGEQFNTFTATSFEGNLGLCGFQVLKEC

>POPTR_0012s00570.1
DLSNNKFTGEIPELIGKLKAVQQLNFSHNSLTGHIQSSIGMLTYLESLDLSSNLFTGRIPVQLADLTFLGV
LNLSHNQLEGPIPSGKHFNTFNASSFEGNLGLCGFPMPKEC

>POPTR_0012s03280.1
DLSNNSFTGEISKVIGKLKALQQLNLSHNFLTGHIQSLLGNLTNLESLDLSSNLLTGRIPMQMAHLTFLAI
LNLSHNQLEGPIPSGKQFDTFDASSFEGNLGLCGFQVLKEC

>POPTR_0012s00250.1
DFSNNNFIGEISKVIGKLKALQQLNLSHNSLTGHIQSSLGMLTYLESLDLSSNLLTGRIPVQLADLTFLGV
LNLSHNHLEGPMPIGKQFNTFNASSFEGNLGLCGFPMPKEC

>POPTR_0012s00820.1
DLSNNNFTGEIPKVIEKLKALQQLNLSHNSLTGHIQSSLGNLTNLESLDLSSNLLTGRIPMQLEGLTFLAI
LNLSHNQLEGPIPSGEQFNTFNANLFEGNLGLCGFQVLKEC

>POPTR_0012s00800.1
DLSNNSFTGEIPKVIGKLKALQQLNLSHNFLTGHIQSSLGNLTNLESLDLSSNLLTGRIPMQMAHLTFLAT
LNLSHNQLEGPIPSGEQFNTFDARSFEGNSGLCGFQVLKEC

>POPTR_0012s00500.1
DLSNNSFIGEIPKMIGKFKAVQQLNLSHNSLTGHIQSSFGMLTYLESLDLSSNLLTGRIPVQLTDLTFLAV
LDLSHNKLEGPVPGGKQFNTFNASSFEGNLDgGFPMPKEC

>POPTR_0012s00560.1(modif)

DLSNNNFIGEISKVIGKLKAIQQLNLSHNSLTGHIQSSIGMLTDLESLDLSSNFLTGRIPVQLADLTFLGV
LNLSHNQLEGPIPSRNQFSTFNASSFEGNLGLCGLPMPKEC

>POPTR_0012s00240.1
DLSNNNFIGEISKVIGKLKALQQLNLSHNSLTGHIQSSIGMLTYLESLDLSSNFLTGRIPVQLADLTFLGV
LNLSHNQLEGPIPSGNQFNTFNASSFEGNLRLCGFPMPNC

>POPTR_0012s03210.1
DLSNNNFTGEIPKVIGKLKALQQLNLSHNSLTGHIQSSLGILANLESLDLSSNLLTGRIPMQLEGLTFLAI
LNLSHNQLEGPIPSGEQFNTFNASSFEGNLGLCGFQVLKEC

>POPTR_0011s07890.1
DLSKNNFIGEIPKVIGKLKALQQLNLSHNSLTGQIQSSLGNLTNLESLDLSSNLLTGRIPTQLAGLTFLAI
LNLSHNQFEGRIPSGEQFNTFTATSFEGNLGLCGFQVLKEC

>POPTR_0011s00940.1
DLSNNSFHGEIPEEIRILKSLIVLTLSHNNFLGQIPSSLSDLTELESLDLSSNHLSGEIPPQLSRLTFLAV
MNLSYNHLEGRIPQGNQFLTFPSSSYEGNPRLCGPPLTRKC

>POPTR_0011s14440.1
DLSNNLFEGEIPEEIGDHKLLDVLNMSRNNLIGEIPTSLSKLTLLESLDLSKNKLTGAIPMQLISLTFLSV
LNLSYNRLEGKIPVGNQFSTFTSDSYQENLGLCGFHVQKC

>POPTR_0011s07880.1
DLSNNNFTGEIPKVIGKLKALHQLNLSHNSLTGHIQSSLGNLTNLESLDLSSNLLTGRIPTQLGGLTFLAV
LNLSHNQLEGRIPSGEQFNTFTATSFEGNLGLCGFQVLKEC

>POPTR_0011s10640.1
DLSCNKFTGKIPESLGKLKSLKQLNLSHNSLIGFIQPSLGNLTNLESLDLSSNLLAGRIPQELVDLTFLQV
LNLSYNQLEGPIPLGKQFNTFENGSYEGNLGLCGFPLQVKC

>POPTR_0011s10651.1
DLSCNKFTGKIPESLGKLKSLIQLNLSHNSLIGYIQPSLGNLTNLESLDLSSNLLAGRIPQQLVDLKYLQV
LNLSYNQLEGPIPVGKQFNTFENGSYEGNLGLCGFPLQVKC

>POPTR_0011s00920.1
DLSNNSFHGEIPEEIRILKSLIVLTLSHNNFLGQIPSSLSDLRELESLDLSSNLLSGEIPPQLSRLTFLAV
MNLSYNHLEGRIPQGNQFDIFPDSSYEGNPRLCGFPLKRKC

>POPTR_0011s10610.1
DLSCNKFTGKIPESLGKLKSLIQLNLSHNSLVGYIQPSLGNLTNLESLDLSSNLLAGRIPPQLVDLTFLQV
LNLSYNQLEGPIPQGKQFNTFENGSYEGNLGLCGLPLQVKC

>POPTR_0011s07920.1
DLSNNNFTGEIPKVIGKLKALQQLNLSHNSLTGHIQSSLGILTNLESLDLSSNLLTGRIPMQLEGLTFLAI
LNLSHNQFEGRIPSGEQFNTFTATSFEGNLGLCGFQVLKEC

>POPTR_0011s07850.1
DLSNNNFTGEIPKAIGKLKALHQLNLSYNFLTGHIQSSLENLNNLESLDLSSNLLTGRIPTQLGGLTFLAI
LNLSHNRLEGRIPSGKQFNTFNASSFEGNWGLCGFQVLKQC

>POPTR_0081s00210.1
DLSCNKFTGKMPESLGKLKSLIQLNLSHNSLIGYIQPSLGNLTNLESLDLSSNLLAGRIPPQLVDLTFLQV
LNLSYNHLEGPIPQGKQFSTFENGSYEGNLGLCGLPLQVKC

>POPTR_0081s00240.1
DLSCNKFTGKIPESLGKLKSLIQLNLSHNSLIGYIQPSLGNLTNLESLDLSSNLLAGRIPPQLVDLTFLEV
LNLSYNQLEGPIPQGKQFHTFENGSYEGNLGLCGLPLQVKC

```
>POPTR_0015s05580.1
DFAGNKLTGEIPEEITSLLQLVAMNLSGNNLTGGIPLKIGQLKQLESLDLSGNQLSGVIPSSTASLSFLSY
LNLSYNNLSGKIPSGTQLQSFNASAFAGNLALCGLPVTHKC

>POPTR_0015s05870.1
NFARNKLIGEIPEEITGLLLLLALNLSGNNLTGEIPQKIWQLKQLESLDLSGNQLSGVIPITMADLNFLAF
LNLSNNHLSGRIPSSTQLQGFNASQFTGNLALCGKPLLQRC

>POPTR_0015s05920.1
NLARNKLIGEIPEEITGLLLLLALNLSGNTLSGEIPQKIGQLKQLESLDLSGNQLSGVIPITMADLNFLAF
LNLSNNHLSGRIPSSTQLQGFNASQFTGNLALCGKPLLQKC

>POPTR_0015s05830.1
DFAGNNLSGEIPEEITGLLGLVALNLSRNNLTGVIPQTIGLLKSLESLDLSGNQFSGAIPVAMGDLNFLSY
LNVSYNNLSGQIPSSTQLQSFDASAFKGNPALCGLPVTHKC

>POPTR_0015s05940.1
NFARNKLIGEIPEEITGLLLLVALNLSGNNLTGEIPQKIGQLKQLESLDLSGNQLSGVIPITMADLNFLAF
LNLSNNHLSGRIPSSTQLQGFNASQFTGNLALCGQPLLQKC

>POPTR_0015s05890.1
DFSGNKLQGEIPEEITGLLLLVALNLSGNNLTGEIPQKIGQLKQLESLDLSGNQLSGVIPITMADLTFLSY
LNLSNNHLSGRIPSSTQLQGFNASQFTGNHALCGQPLLQKC

>POPTR_0001s03540.1
DLSSNNLSGEIPDGIAKLEGLVSLHLSNNRLTGIIPPRIGLMRSLESLDLSTNQLSGGLPNGLRDLNFLSS
LNVSYNNLSGKIPLSTQLQTFDNNSFVANAELCGKPLSNEC

>POPTR_0001s42850.1
DLSNNRFEGEIPEMICDLKLLQVLNLSRNNLVGEIPLSLSKLAKLESLDLSQNKLTGEIPMQLTDLTFLSV
LNLSYNRLVGRIPVANQFLTFANDSYGGNLGLCGFPLSRKL

>POPTR_0001s41110.1
DLSSNAFQGDIPQSIGTLEKVNALNLSNNHLSGDIPSVLGNLANLESLDLSQNMLSGEIPQYLTQLTFLAY
FNVSHNQLEGPIPQGKQFNTFDNSSYEGNSGLYMKHLPKKS

>POPTR_0001s39970.1
DLSSNRFEGQIPKEVGLLSSLIVLNISRNSVTGQIPSSLGNLTALESLDLSSNGLGGGIPSQLTRLTFLAV
LNLSYNQLVGPIPHGSQFDTFQNDSYVGNLRLCGFPLSVKC

>POPTR_0001s42840.1
DLSNNLFEGEIPEVIGDLKLLEVLNLSTNNLIGEIPLSLSKLTLLESLDLSKNKLIGEIPMKLLSLTFLSV
LNLSYNRLEGKIPIGNQFSTFANDSYEGNIGLCGFPLSKKC

>POPTR_0001s42860.1
DLSNNRFEGEIPEMICDLKLLQVLNLSRNNLVGEIPTSLSKLAKLESLDLSQNKLTGEIPMQLTDLTFLSV
LNLSYNRLVGRIPVANQFLTFANDSYGGNLGLCGFPLSRKC

>POPTR_0001s27000.1
DLSINSLNGEFPDQITKLVGLVTLNLSKNQVSGHVPDNISSLRQLSSLDLSSNRLSGAIPSSLPALSFLSY
LNLSNNNLSGMIPYRGQMTTFEASSFSGNPGLCGPPLVLQC

>POPTR_0001s01060.1
DLSNNLFEGEIPEKIGDLDLLYVLNLSNNHLTGQIPSSFGKLKELGSLDLSENRLSGTIPQQLTTLTFLSV
LKLSQNLLVGEIPQGNQFGTFTSAAFEGNIGLCGPPLTKTC

>POPTR_0027s00410.1
```

DLSNNNFVGAIPPEFGDLSKILSLNLSHNNLTGSIPATFSNLKQIESLDLSYNNLNGVIPPQLTDITTLEV
FSVAHNNLSGNTPERKYQFGTFDESCYEGNPFLCGPPLRNNC

>POPTR_0027s00350.1
DLSNNNFIGAIPPEFGNLSKILSVNLSHNNLTGSIPATFSNLMHIESLDLSYNNLNGAIPPQFTEVTTLEV
FSVAHNNLSGKTPERIYQFGTFDESCYEGNPFLCGPPLPNNC

>POPTR_0010s11720.1
DLSRNRFVGEIPNQLMNLLELRNLNLSRNNFKGQIPWKIGDLRQLQSLDLSRNEISGLIPTSLSQLNFLSA
LNLSFNKLSGRIPSGNQLQTLDDKSIYAGNSGLCGFPLDDC

>POPTR_0010s11730.1
DLSRNRFVGEIPNQLMNLLELRNLNLSRNNFKGQIPWKIGDLRQLQSLDLSRNEISGLIPTSLSQLNFLSA
LNLSFNKLSGRIPSGNQLQTLDDKSIYAGNSGLCGFPLDDC

>POPTR_1137s00200.1
GPLPTQFPSEKSKNDRKVKAVQQLNLSHNSLTGHIQSSFGMLTYLESLDLSSNLLTGRIPVQLADLTFLAV
LDLSHNKLEGPVPGGKQFNTFNASSFEGNLDLCGFPMPKEC

>POPTR_0008s18640.1
DFSFNNFTGEIPPEIGNLSMIKALNLSHNSLTGPIPPTFWNLKEIESLDLSYNKLDGEIPPRLIELFSLEV
FNVAHNNLSGKIPARVAQFATFDESCYKDNPFLCGEPLPKIC

>POPTR_0016s12810.1
DFSSNNFEGPIPDAIGKFNALYVLNLSHNVLTGQIPSSLGNLSQLESLDLSSNQLSGQIPAQLTSLTFLSV
LNLSYNRLVGRIPTGNQFLTFSSDSFEGNQGLCGPPLKLAC

>POPTR_0016s12800.1
DFSSNNFEGPIPDAIGQFNVLYVLNLSHNVLTGQIPSSLGNLSQLESLDLSSNQLSGQIPAQLTSLTFLSV
LNLSYNRLVGRIPTGNQFLTFSSDSFEGNQGLCGPPLILSC

>POPTR_0016s13460.1
DLSSNGFEGGIPEVLGDLKELHLLNLSNNFLSGGIPPSLSNLKELEALDLSQNKLSGEIPVKLAQLTFLEV
FNVSHNFLSGPIPRGNQFGTFENTSFDANPGLCGEPLSKEC

>POPTR_0016s13480.1
DLSSNRFEGGIPDALGDLKELYLLNLSNNFLTGRIPPSLSNLKGLEALDLSQNKLSGEIPVQLAQLTFLAV
FNVSHNLLSGPIPRGNQFETFDSTSFDANSGLCGKPLSKKC

>POPTR_0016s13470.1
DLSSNRFEGGIPDALGDLKELYLLNLSNNFLTGRIPPSLSNLKGLEALDLSQNKLSGEIPVQLAQLTFLAV
FNVSHNLLSGPIPRGNQFETFDSTSFDADSGLCGKPLSKKC

>AT2G15080.1-RLP19
DFSGNKFEGEIPKSIGLLKELHVLNLSNNALSGHIASSMGNLMALESLDVSQNKLSGEIPQELGKLTYLAY
MNFSHNQLVGLLPGGTQFQTQKCSSFEDNHGLYGPSLEKIC

>AT2G32680.1-RLP23
DFSGNRLEGQIPESIGLLKALIAVNISNNAFTGHIPLSMANLENLESLDMSRNQLSGTIPNGLGSISFLAY
INVSHNQLTGEIPQGTQITGQSKSSFEGNAGLCGLPLKESC

>AT2G25470.1-RLP21
DLSNNELSGVIPTELGDLLKLRTLNLSHNSLLGSIPSSFSKLIDVESLDLSHNMLQGSIPQLLSSLTSLAV
FDVSSNNLSGIIPQGRQFNTFEEESYLGNPLLCGPPTSRSC

>AT2G25440.1-RLP20
DFSRNLLEGNIPESIGLLKALIALNLSNNAFTGHIPQSLANLKELQSLDMSRNQLSGTIPNGLKQLSFLAY
ISVSHNQLKGEIPQGTQITGQLKSSFEGNVGLCGLPLEERC

>AT2G33020.1-RLP24
DFSGNRLQGQIPESIGLLKALIALNLSNNAFTGHIPLSFANLMNLESLDMSGNQLSGTIPNGLGSLSFLVY
ISVAHNKLKGEIPQGTQITGQIKSSFEGNAGLCGLPLQETC

>AT2G33060.1-RLP27
DFSGNKLEGQIPESIGLLKALIALNLSNNAFTGHIPLSLANVTELESLDLSRNQLSGTIPNGLKTLSFLAY
ISVAHNQLIGEIPQGTQITGQSKSSFEGNAGLCGLPLQGSC

>AT2G33050.1-RLP26
DFSGNKLEGQIPESIGLLKELIALNLSNNAFTGHIPMSLANVTELESLDLSRNQLSGNIPRELGSLSFLAY
ISVAHNQLKGEIPQGPQFSGQAESSFEGNVGLCGLPLQGSC

>AT2G32660.1-RLP22
DFSGNKLEGEIPESIGLLKTLIALNLSNNSFTGHIPMSFANVTELESLDLSGNKLSGEIPQELGRLSYLAY
IDVSDNQLTGKIPQGTQIIGQPKSSFEGNSGLCGLPLEESC

>AT4G13920.1-RLP50
DVSGNRLEGDIPESIGILKELIVLNMSNNAFTGHIPPSLSNLSNLQSLDLSQNRLSGSIPGELGELTFLAR
MNFSYNMLEGPIPQGTQIQSQNSSSFAENPGLCGAPLQKKC

>AT4G13880.1-RLP48
DVSGNRLEGDIPESIGILKELIVLNMSNNAFTGHIPPSLSNLSNLQSLDLSQNRLSGSIPPELGKLTFLEW
MNFSYNRLEGPIPQATQIQSQNSSSFAENPGLCGAPFLNKC

>AT4G13810.1-RLP47
DVSGNRLEGDIPESIGLLKEVIVLSMSNNAFTGHIPPSLSNLSNLQSLDLSQNRLSGSIPGELGKLTFLEW
MNFSHNRLEGPIPETTQIQTQDSSSFTENPGLCGAPLLKKC

>AT1G71390.1-RLP11
DFSENRIYGEIPESIGCLEELRLLNLSGNAFTSDIPRVWENLTKLETLDLSRNKLSGQIPQDLGKLSFLSY
MNFSHNRLQGPVPRGTQFQRQRCSSFLDNHRLYGLEDIC

>AT1G74170.1-RLP13
DLSENELSGEIPVELGGLVELEALNLSHNNLSGVILESFSGLKNVESLDLSFNRLQGPIPLQLTDMISLAV
FNVSYNNLSGIVPQGRQFNTFETQSYFGNPLLCGKSIDISC

>AT1G74180.1-RLP14
DLSSNELSGVIPAELGDLSKLRALNLSRNLLSSSIPANFSKLKDIESLDLSYNMLQGNIPHQLTNLTSLAV
FNVSFNNLSGIIPQGGQFNTFNDNSYLGNPLLCGTPTDRSC

>AT1G71400.1-RLP12
DFSGNKINGNIPESLGYLKELRVLNLSGNAFTSVIPRFLANLTKLETLDISRNKLSGQIPQDLAALSFLSY
MNFSHNLLQGPVPRGTQFQRQKCSSFLDNPGLYGLEDIC

>AT1G74190.1-RLP15
DLSENELSGEIPVEFGGLLELRALNLSHNNLSGVIPKSISSMEKMESFDLSFNRLQGRIPSQLTELTSLSV
FKVSHNNLSGVIPQGRQFNTFDAESYFGNRLLCGQPTNRSC

>AT1G47890.1-RLP7
DLSGNQLHGKIPDSIGLLKELRILNMSSNGFTGHIPSSLANLKNLESLDISQNNISGEIPPELGTLSSLAW
INVSHNQLVGSIPQGTQFQRQKCSSYEGNPGLNGPSLENVC

>AT1G07390.1-RLP1
DLSSNELSGEIPIEIGDLQNIRSLNLSSNRLTGSIPDSISKLKGLESLDLSNNKLDGSIPPALADLNSLGY
LNISYNNLSGEIPFKGHLVTFDERSYIGNAHLCGLPTNKNC

>AT1G58190.1-RLP9

DFSSNELIGEIPRELGDFQRIRALNLSHNSLSGLVPESFSNLTDIESIDLSFNVLHGPIPHDLTKLDYIVV
FNVSYNNLSGLIPSQGKFLSLDVTNYIGNPFLCGTTINKSC

>AT3G05660.1-RLP33
DFSGNKFEGEIPRSIGLLKELHILNLSSNGFTGHIPSSMGNLRELESLDVSRNKLSGEIPQELGNLSYLAY
MNFSHNQLVGQVPGGTQFRTQSASSFEENLGLCGRPLEEC

>AT3G23110.1-RLP37
DFSGNRFSGHIPRSIGLLSELLHLNLSGNAFTGNIPPSLASITKLETLDLSRNNLSGEIPRGLGKLSFLSN
INFSHNHLEGLVPQSTQFGSQNCSSFMGNPRLYGLDQIC

>AT3G23010.1-RLP36
DFSGNRFSGHIPGSIGLLSELRLLNLSGNAFTGNIPPSLANITNLESLDLSRNNLSGEIPISLGKLSFLSN
TNFSYNHLEGLIPQSTQFATQNCSSFLGNLGLYGFREIC

>AT3G25010.1-RLP41
DLSGNRLEGEIPESIGLLKALIALNLSNNAFTGHIPLSLANLVKIESLDLSSNQLSGTIPNGLGTLSFLAY
VNVSHNQLNGEIPQGTQITGQPKSSFEGNAGLCGLPLQQRC

>AT3G05370.1-RLP31
NFSGNRFSGNIPESIGLLKELRHLNLSSNAFTGNIPQSLANLMKLEALDLSLNQLSGQIPQGLGSLSFMST
MNFSYNFLEGPVPKSTQFQGQNCSAFMENPKLNGLEEIC

>AT3G11080.1-RLP35
DFSGNKFEGEIPKSIGLLKELLVLNLSNNAFGGHIPSSMGNLTALESLDVSQNKLTGEIPQELGDLSFLAY
MNFSHNQLAGLVPGGTQFRRQNCSAFENNLGLFGPSLDEVC

>AT3G11010.1-RLP34
DFSGNKFEGEIPKSIGLLKELHVLNLSNNAFTGHIPSSIGNLTALESLDVSQNKLYGEIPQEIGNLSLLSY
MNFSHNQLTGLVPGGQQFLTQRCSSFEGNLGLFGSSLEEVC

>AT3G05650.1-RLP32
DFSENKLEGEIPRSIGLLKELHVLNLSSNAFTGHIPSSMGNLRELESLDVSQNKLSGEIPQELGNLSYLAY
MNFSHNQLGGLVPGGTQFRRQNCSSFKDNPGLYGSSLEEVC

>AT3G53240.1-RLP45
DLSSNELSGNIPEELGDLKRVRSLNLSRNSLSGSIPGSFSNLRSIESLDLSFNKLHGTIPSQLTLLQSLVV
FNVSYNNLSGVIPQGKQFNTFGEKSYLGNFLLCGSPTKRSC

>AT3G24982.1-RLP40
DFSGNRLEGEIPESIGLLKALIALNLSNNAFTGHIPLSFANLKKMESLDLSSNQLSGTIPNGLRTLSFLAY
VNVSHNQLIGEIPQGTQITGQPKSSFEGNAGLCGFPLQESC

>AT3G24900.1-RLP39
DFSGNRLEGEIPESIGLLKALIALNLSNNAFTGHIPLSLANLKKIESLDLSSNQLSGTIPNGIGTLSFLAY
MNVSHNQLNGEIPQGTQITGQPKSSFEGNAGLCGLPLQESC

>AT3G28890.1-RLP43
DFSGNKFEGEIPKSIGLLKELLVLNLSNNAFTGHIPSSMGKLTALESLDVSQNKLYGEIPQEIGNLSFLSC
MNFSHNQLAGLVPGGQQFLTQPCSSFEDNLGLFGSTLEEDC

>AT3G25020.1-RLP42
DLSGNRLEGEIPESLGLLKALIALNLSNNAFTGHIPLSLANLKKIESLDLSSNQLSGTIPNGLGTLSFLAY
MNVSHNQLNGEIPQGTQITGQPKSSFEGNAGLCGFPLQESC

>AT3G05360.1-RLP30
DFSGNRFFGNIPESVGLLKELRLLNLSGNSFTSNIPQSLANLTNLETLDLSRNQLSGHIPRDLGSLSFLST
MNFSHNLLEGPVPLGTQFQSQHCSTFMDNLRLYGLEKIC

>AT5G49290.1-RLP56
DLSSNELSGVIPAELGDLFKLRALNLSHNFLSSHIPDSFSKLQDIESLDLSYNMLQGSIPHQLTNLTSLAI
FNVSYNNLSGIIPQGKQFNTFDENSYLGNPLLCGPPTDTSC

>AT5G27060.1-RLP53
DFSGNRFEGEIPKSIGLLKELLVLSLSNNAFSGHMPSSMGNLTALESLDVSKNKLTGEIPQELGDLSFLAY
MNFSHNQLAGLVPGGQQFLTQNCSAFEDNLGLFGSSLEEVC

>AT5G25910.1-RLP52
DFSGNKFEGEIPRSVGLLKELHVLNLSNNGFTGHIPSSMGNLIELESLDVSQNKLSGEIPPELGKLSYLAY
MNFSQNQFVGLVPGGTQFQTQPCSSFADNPRLFGLSLERVC

>AT5G40170.1-RLP54
DFSGNSFEGQIPESIGDLKSLIVLDLSNNSFTGRIPSSLAKLKQLESLDLSQNRISGNIPQELRELTFLGY
VNMSHNRLTGQIPQSTQVGGQPKSSFEGNINLCGLPLQESC

>AT1G45616.1-RLP6
DFAGNKIQGKIPESVGILKELHVLNLSSNAFTGHIPSSLANLTNLESLDISQNKIGGEIPPELGTLSSLEW
INVSHNQLVGSIPQGTQFHRQNCSSYEGNPGIYGSSLKDVC

>AT2G33080.1-RLP28
DFSGNRLEGQIPKSIGLLKELIALNLSNNAFTCHIPLSLANATELESLDLSRNQLSGTIPNGLKTLSFLAY
INVSHNKLKGENHKEHRLLGNINPPLKGMQGFVVFLWRKLA

>AT3G23120.1-RLP38
DFSGNRFSGHIPRSIGLLSELLHLNLSGNAFTGNIPPSLANITNLETLDLSRNNLSGEIPRSLGNLSFLSN
INFSHNHLQGFVPRSTQFGTQNCSSFVGNPGLYGLDEIC

>AT1G54470.2-RPP27
DLSSNELSGVIPAELGSLSKLRVMNLSCNFLSSSIPSSFSNLKDIESLDLSHNMLQGSIPQQLTNLSSLVV
FDVSYNNLSGIIPQGRQFNTFDEKSYLGNPLLCGPPTNRSC

>POPTR_0018s14150.1
DFSCNNFTGGIPPEIGNLNMIKVLNLSHNSLTGPIPPTFSNLKEIESLDLSYNKLDGEIPPRLTELFSLEV
FSVAHNNLSGNTPVRVAQFATFEENCYKDNPFLCGEPLPKIC

>AT4G04220.1-RLP46
DLSKNKLHGEIPTSLGNLKSLKVLNLSNNEFSGLIPQSFGDLEKVESLDLSHNNLTGEIPKTLSKLSELNT
LDLRNNKLKGRIPESPQLDRLNNPNIYANNSGICGMQIQVPC

>AT1G17250.1-RLP3
YIRRNNLKGSIPIEVGQLKVLHVLELSHNYLSGIIPHELSKLTSLERLDLSNNHLSGRIPWSLTSLHYMSY
FNVVNNSLDGPIPTGSQFDTFPQANFKGNPLLCGGILLTSC

>AT1G17240.1-RLP2
YIRRNNLTGSIPVEVGQLKVLHILELLGNNLSGSIPDELSNLTNLERLDLSNNNLSGSIPWSLTNLNFLSY
FNVANNSLEGPIPSEGQFDTFPKANFEGNPLLCGGVLLTSC

>AT1G65380.1-RLP10-CLV2
DLSDNLLHGEIPEALFRQKNIEYLNLSYNFLEGQLPRLEKLPRLKALDLSHNSLSGQVIGNISAPPGLTLL
NLSHNCFSGIITEKEGLGKFPGALAGNPELCVETPGSKC

Figure S1. Poplar *LRR-RLP* and *SPUF* genes with notable 517 **tissue- or stress-specific expression patterns.**

Available expression profiles of 47/82 LRR-RLP and 36/87 SPUF genes have been extracted from the PopGenIE portal. Weakly-expressed and non-regulated *LRR-RLP* and *SPUF* genes (42 and 34, respectively) have been grouped in a single profile. Only five LRR-RLPs and two SPUFs presenting high and regulated expression levels are individually represented. The asterisks indicate the tissues or conditions where the genes are strongly expressed or induced.

Figure S2. Wounding effect in poplar leaf disks compared with entire leaves.

The average expression values of the six conditions in the wounded and non-wounded dataset are presented as a dot plot with log-scale axes. All expression values are presented in Table S1C. The red rectangle indicates the wound-induced genes annotated in Table S1C and briefly discussed in the text.

Figure S3. Gene expression profile of *RISP* and its associated *LRR-RLP* during the interaction between *Populus trichocarpa* cultivar "Beaupré" and the rust fungus *Melampsora larici-populina* isolates 93ID6 (incompatible interaction) and 98AG31 (compatible interaction).

Samples were harvested before inoculation (0 hours post inoculation, hpi) and after 6, 12, 18, 24, 30, 36, 42, 60, 72 and 96 hpi. RNAseq reads were normalized according to the library size and overrepresented (positive values) and underrepresented (negative values). Transcripts are shown as log2 fold changes relative to the mean expression measured across all 22 samples. Error bars: standard error."

Figure S4. The Risp gene is physically associated and shares a highly conserved promoter region with a LRR-RLP gene in the poplar genome.

(A) Schematic representation of the location of RISP (red) and the associated Leucine-Rich Repeat Receptor-Like Protein (LRR-RLP) gene (blue) on *Populus trichocarpa* genomic sequence. The 400-nucleotide promoter regions with 90% of nucleotide identity are identified with light green boxes. **(B)** Nucleotide alignment of the two conserved promoter regions of Risp (-670 to -1039) and LRR-RLP (-722 to -1122) genes.

Figure S5. SNPs alignment between LRR-RLP580 and LRR-RLP9.

All the single nucleotide polymorphisms (SNPs) observed within the extracellular part of LRR-RLPs 580 and 9 (*i.e.* from domain B to the middle of domain C3) are represented in blue (LRR-RLP 9) and in red (LRR-RLP 580). SNPs profiles confirmed from the cloned cDNA or from genomic DNA are indicated. Numbers read vertically and indicate the position of the SNP.

Table S1. Supplemental information and datasets. (Sheet A) Details about *LRR-RLP* and associated *SPUF* genes identified in poplar and arabidopsis. (Sheet B) SPUF sequences and paralogs in poplar and arabidopsis as well as homologs in other plants. (Sheet C) Oligoarray dataset and wound-induced genes in leaf disks compared with entire leaves. (Sheet D) genes encoding small-secreted proteins of interest identified in the vicinity of arabidopsis *LRR-RLP* genes.

Figure S1

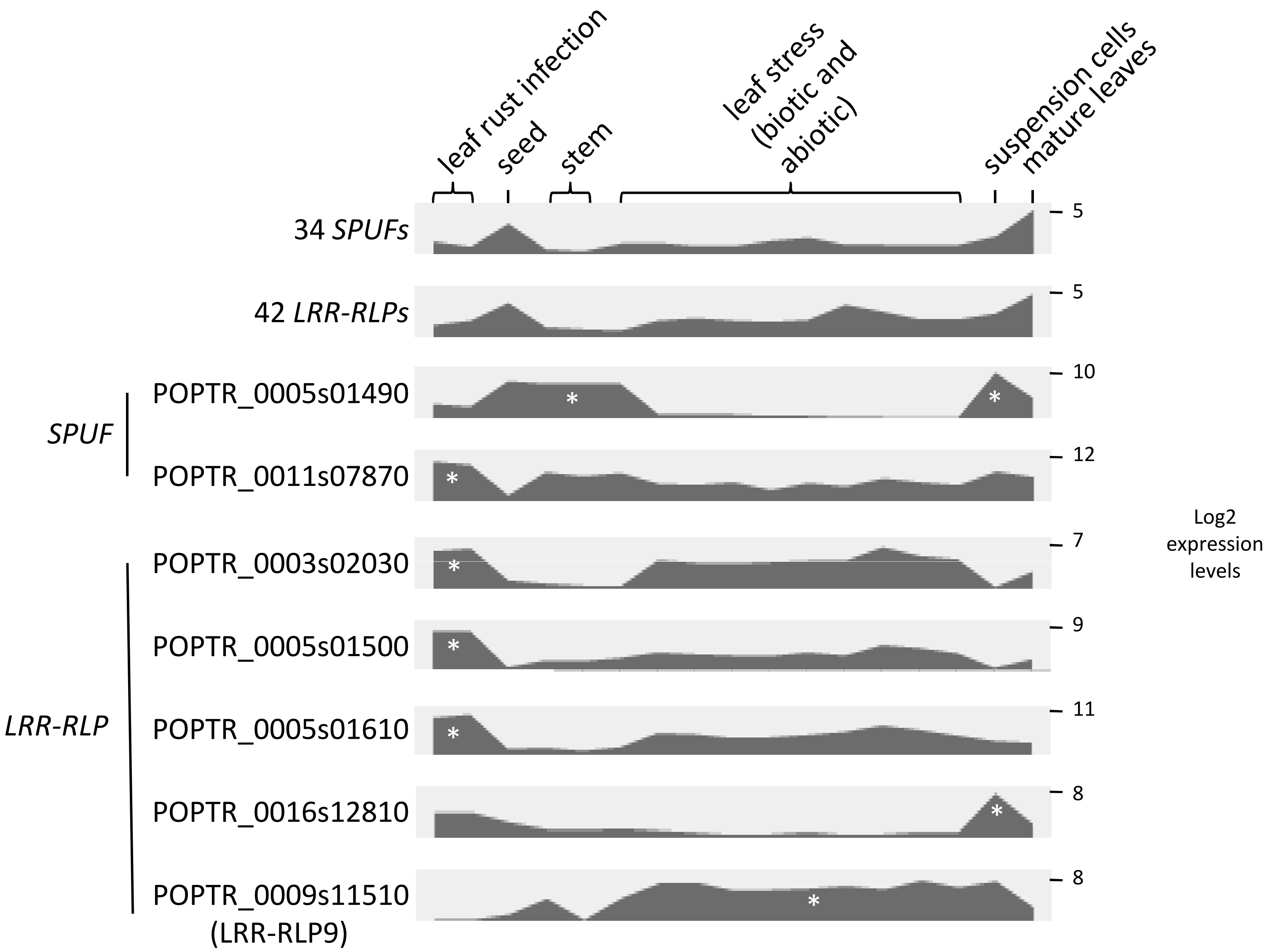
leaf rust infection
seed
stem
leaf stress (biotic and abiotic)
suspension cells
mature leaves
34 SPUFs
42 LRR-RLPs
SPUF
POPTR_0005s01490
POPTR_0011s07870
LRR-RLP
POPTR_0003s02030
POPTR_0005s01500
POPTR_0005s01610
POPTR_0016s12810
POPTR_0009s11510 (LRR-RLP9)
5
5
10
12
7
9
11
8
8
Log2 expression levels

Figure S2

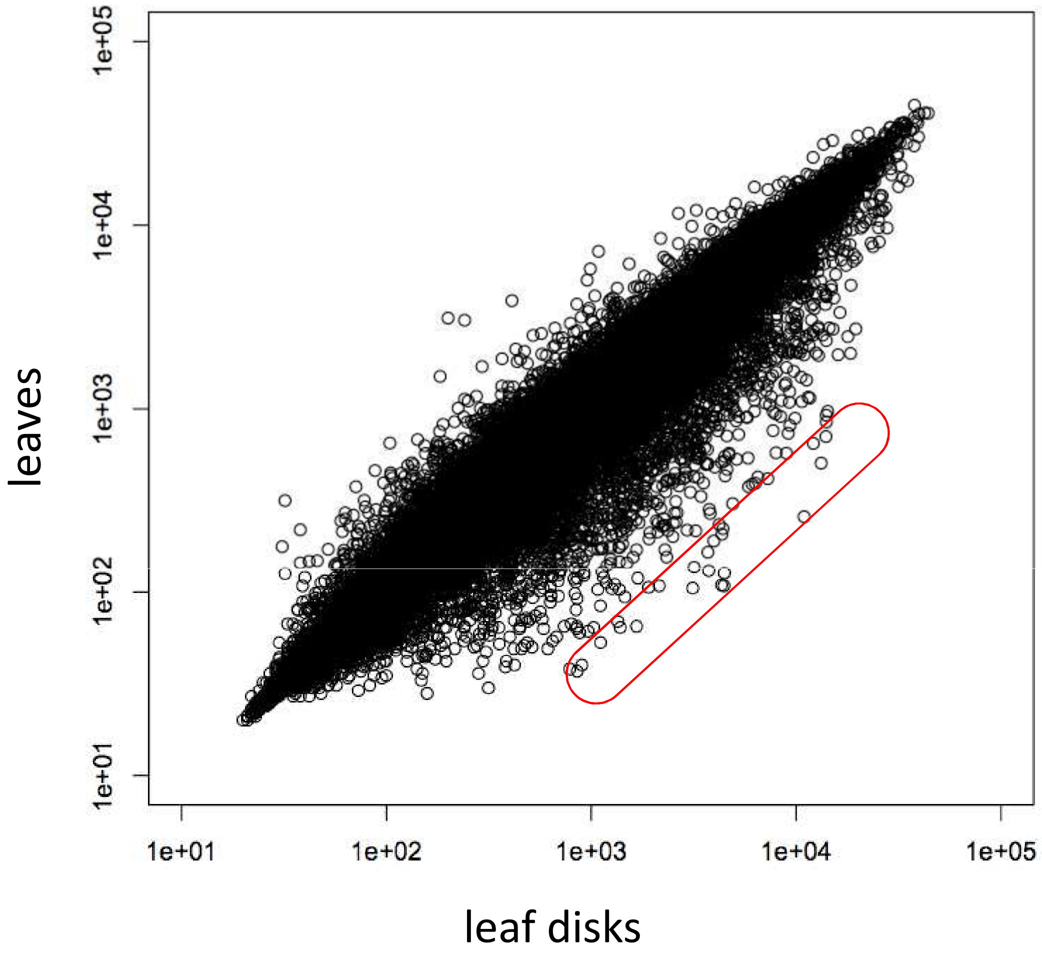

Figure S3

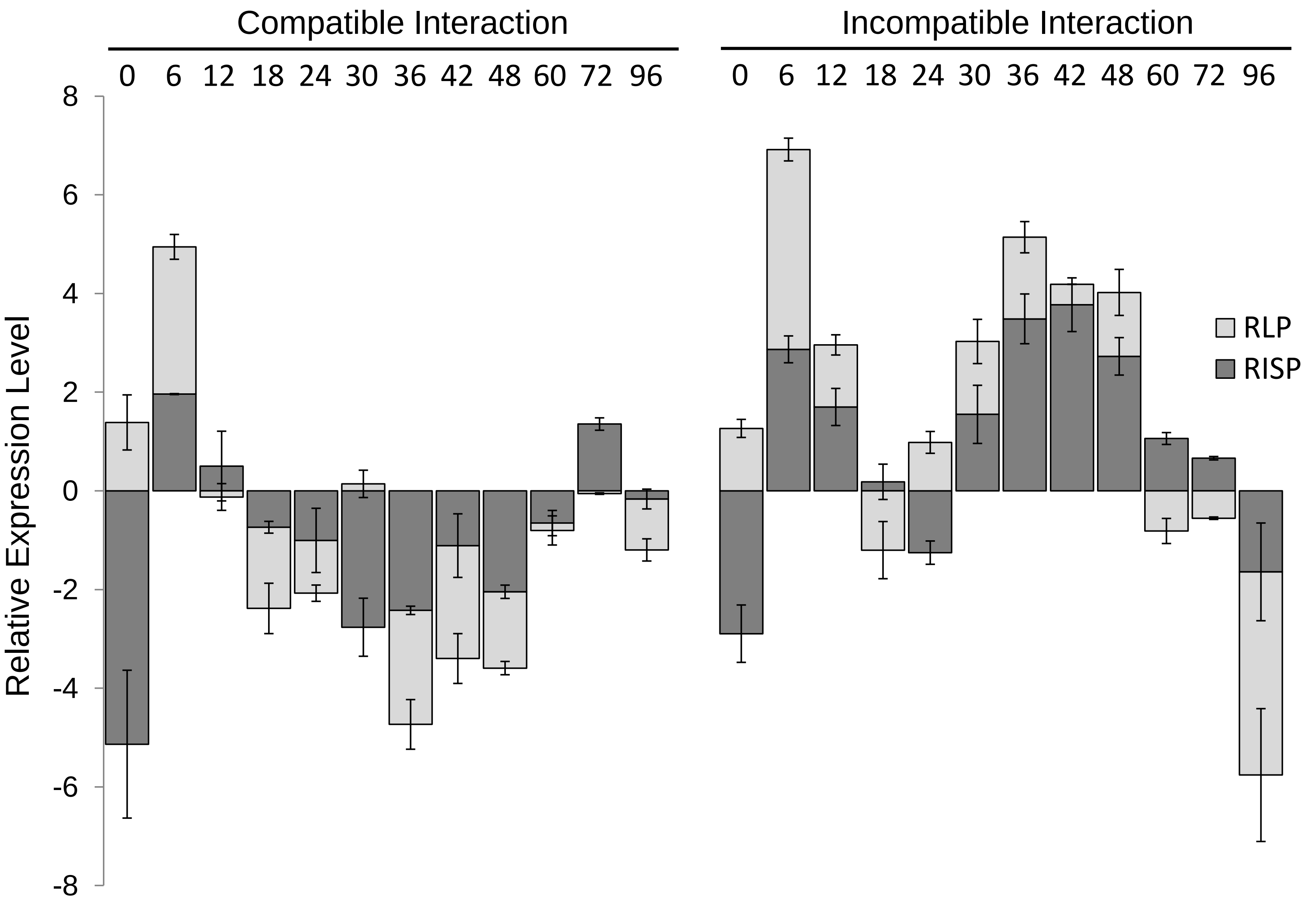

Compatible Interaction
Incompatible Interaction
0 6 12 18 24 30 36 42 48 60 72 96
0 6 12 18 24 30 36 42 48 60 72 96
Relative Expression Level
8
6
4
2
0
-2
-4
-6
-8
RLP
RISP

A

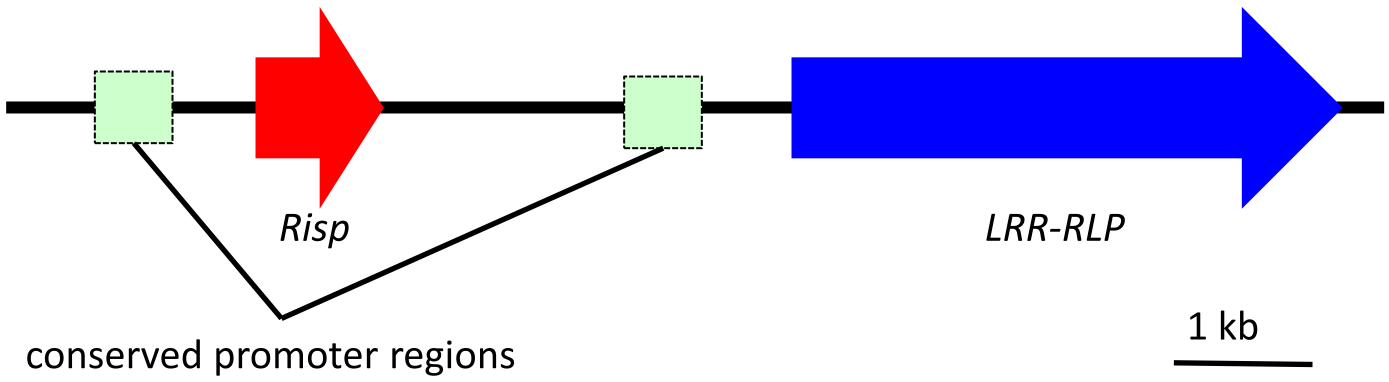

B

```
                    1                                                50                                                100
Risp-671/-1039      GTAATGTCCACACCTTCTACTTCTGGCTCAACAAAGGTTCAATGTTCGACGGACCCTTCCCCTTCTTTAATAAAGACTTGACATGGAGCCACCGCAGCCA
LRR-RLP-722/-1122   GTAAGGTCCACACCTTCTACTTCTGGCTTAACAAAGGTTCAATGTTCGATGGATCCTTCCCCTTCTTTAATAAGGAATTGACTTGGAGCCAACGCAGCCA
Consensus           GTAA.GTCCACACCTTCTACTTCTGGCT.AACAAAGGTTCAATGTTCGA.GGA.CCTTCCCCTTCTTTAATAA.GA.TTGAC.TGGAGCCA.CGCAGCCA

                    101                                              150                                               200
Risp-671/-1039      GGAAGGATCCAACGAGTGTCCAAAGAAAGAATGTCTTAAAATAAACCATTTGAGACCAAGTTGTCCTGTTTCAAGTCAAAAAGGGTCCAGAAGAGCAATG
LRR-RLP-722/-1122   GGAAGGAGCCAACGAGTGTCCGAAGAAAAAATGTCTTAAAATAAACCATTTTAGACCAAGTTGTCCTGTTTCAAGTCAAAAATGGTCCGGAAGAGCAATG
Consensus           GGAAGGA.CCAACGAGTGTCC.AAGAAA.AATGTCTTAAAATAAACCATTT.AGACCAAGTTGTCCTGTTTCAAGTCAAAAA.GGTCC.GAAGAGCAATG

                    201                                              250                                               300
Risp-671/-1039      CCAAAGACTTTAATGCAAGATAGAATTGTATCGTTGGCTGTCGGCCGGAGGAGCCCCGATTTTCAAGTCAAG--------------------------
LRR-RLP-722/-1122   CCAAGACTTTAAATACAAGACAGAATTGTATTGTTGGCTGTCGGCTGGAGGCGCCCCGGTTTTCAAGTCAAGGATGAGGGGCTACACTGGCTCCTCTTTC
Consensus           CCAA....TT.AAT.CAAGA.AGAATTGTAT.GTTGGCTGTCGGC.GGAGG.GCCCCG.TTTTCAAGTCAAG..........................

                    301                                              350                                               400
Risp-671/-1039      -TAAGGAGGCTTTTTTT---TATCTATTTATTTATTTTTTAAGCTCAAATACTTCTTGGGGGAGTTGATCCCGTTTTGCAGGTGCAAGGAACTTACTACC
LRR-RLP-722/-1122   GTAAGGAGGCTTTTTTTATCTATTTATTTATTTATTTTATTAGCTCAAATACTTCTAGGAGGATTTGAACCCGTTTTGCAGGTGCAAGGAACTTATTACC
Consensus           .TAAGGAGGCTTTTTTT...TAT.TATTTATTTATTTT.T.AGCTCAAATACTTCT.GG.GGA.TTGA.CCCGTTTTGCAGGTGCAAGGAACTTA.TACC
```

Figure S5

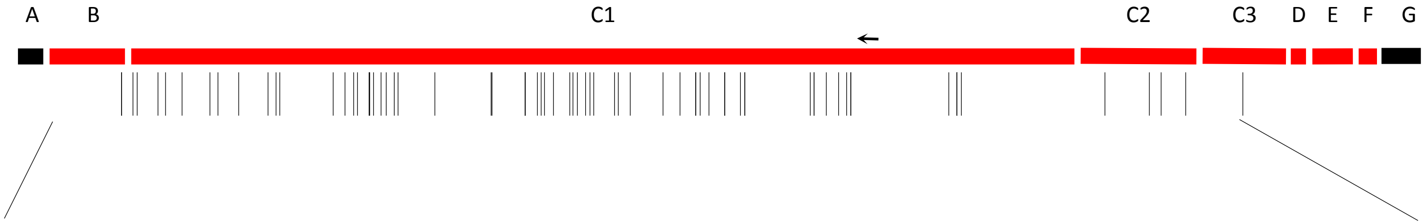


```
                      00000000000000000000001111111111111111111111111111111111111222222222222
                      00111123336666666678990000111111222223444444555566777788888111114566689
                      79016920160245888914896669013789133892148899155900667703566022238928826
                      53644010990511567696903573391596902876095907469958252422913623764712937

LRR-RLP9              GCAGTGTCGCTATCAGAGCACGACTTTCTTGGCTCTAAGGCGTAGTAAAGCTCTGAAGCTGATGTTTTCTT
LRR-RLP580            CTGACTATATCTCTGACCTCGTGTCAATCGATTCACGTCCTTATACCGTTTATGAGGATCAGCTCCCAAAA

Chimera (cloned from cDNAs)
                      .TGACTATATCTCTGACCTACGACTTTCTTGG.....AGGCGTAGTAAAGCTCTGAAGCCAGCTCCCAAAA

Chimera (amplicon from gDNA)
                      .......................................GCGTAGTAAAGCTCTGAAGCCAGCT.......
```
